# Chemical Composition, Antibacterial Activity and *In Vitro* Anticancer Evaluation of *Ochradenus baccatus* Methanolic Extract

**DOI:** 10.3390/medicina59030546

**Published:** 2023-03-10

**Authors:** Weam M. A. Khojali, Weiam Hussein, Mohammed Khaled Bin Break, Ahmed Alafnan, Bader Huwaimel, Nasrin E. Khalifa, Wafa F. S. Badulla, Raghad Abdulkarem Alshammari, Lama Khalid Alshammari, Rehab Aladham Raji Alshammari, Sara Mohsen Albarak, Enas Hmdan Alrkad, Tooba Mahboob, Hisham Alshammari

**Affiliations:** 1 Department of Pharmaceutical Chemistry, College of Pharmacy, University of Hail, Hail 2240, Saudi Arabia; 2 Department of Pharmaceutical Chemistry, College of Pharmacy, Omdurman Islamic University, Al Khartoum 14415, Sudan; 3 Department of Pharmaceutical Chemistry, Faculty of Pharmacy, Aden University, Aden 6075, Yemen; 4 Department of Pharmacology and Toxicology, College of Pharmacy, University of Hail, Hail 2240, Saudi Arabia; 5 Department of Pharmaceutics, College of Pharmacy, University of Hail, Hail 2240, Saudi Arabia; 6 Department of Pharmaceutics, Faculty of Pharmacy, University of Khartoum, Khartoum 11115, Sudan; 7 Faculty of Pharmaceutical Sciences, UCSI University, KL Campus, Kuala Lumpur 56000, Malaysia; 8 Department of Clinical Pharmacy, College of Pharmacy, University of Hail, Hail 2240, Saudi Arabia

**Keywords:** *Ochradenus baccatus*, phytochemicals, antibacterial, cytotoxicity, Hail

## Abstract

*Background and Objectives: Ochradenus baccatus* belongs to the family Resedaceae. It is widely spread in Saudi Arabia and other countries in Southwest Asia. *O. baccatus* is extensively used in traditional medicine as an anti-inflammatory and antibacterial agent, in addition to being a vital source of food for certain desert animal species. The aim of the present study was to investigate the chemical composition and antibacterial/anticancer activities of *O. baccatus* methanolic extracts collected from Hail, Saudi Arabia. *Materials and Methods:* The *O. baccatus* extracts were obtained by macerating the crude powder in methanol, followed by filtration and evaporation. Liquid chromatography–mass spectrometry (*LC-MS*) was used to analyze the methanolic extracts’ chemical constituents. Broth microdilution assay for minimum inhibitory concentration (MIC) determination was used to assess antimicrobial activity, while the extracts’ anticancer potential was assessed by sulforhodamine B Assay (SRB) assay. *Results:* The results of the antibacterial assay showed that the methanolic extracts from the roots and branches possessed varying degrees of activity against particular bacterial strains, with the highest activity being exerted by the branches’ extract against *Escherichia coli* and *Salmonella typhimurium* (St), demonstrating MIC values of 15.6 µg/mL and 20 µg/mL, respectively. Furthermore, the SRB cell viability assay revealed that only the branches’ extract inhibited the growth of A549 cancer cells, with an IC_50_ value of 86.19 µg/mL. The *LC-MS* analysis of the methanolic extracts from the plant’s roots and branches was then conducted, resulting in the identification of 8 and 13 major chemical constituents, respectively. Azelaic acid, β-amyrin, and phytanic acid are some of the bioactive compounds that were detected in the extracts via *LC-MS*, and they are thought to be responsible for the observed antibacterial/anticancer activity of *O. baccatus* methanolic extracts. *Conclusions:* This study confirmed the antibacterial/anticancer potential of *O. baccatus* methanolic extracts and analyzed their phytochemical constituents. Further isolation and biological screening are warranted to understand the therapeutic potential of *O. baccatus*.

## 1. Introduction

Plants have been used to treat numerous diseases since ancient times and continue to be a major source of therapeutic agents in modern medicine. By the middle of the nineteenth century, it had been reported that at least 80% of all drugs were derived from plants [1]. The expanding use of medicinal plants in the treatment of multiple ailments is related to the fact that plants or their derivatives are regarded as safe and effective treatments with minimal side effects and low costs [2]. *Ochradenus baccatus* (Taily Weed) from the family Resedaceae is widely cultivated in Saudi Arabia, Ethiopia, Tunisia, Egypt, Morrocco, Libya, Pakistan, and other countries in the Middle East. This plant is the most common species of the genus *Ochradenu*s and is considered a dioecious or bisexual shrub [3]. *O. baccatus* is traditionally utilized as an anti-inflammatory and antibacterial agent in folk medicine. Moreover, pharmacological studies on *O. baccatus* had found that its ethanolic and ethyl acetate extracts demonstrated growth inhibitory activity against *S. aureus* and *E. coli,* while its hexane extract inhibited *Candida albicans.* Additionally, the plant was found to be safe, with no adverse effects, on the kidneys and liver of treated mice [4]. The plant’s ethanolic extract was also found to possess anti-inflammatory and free-radical scavenging effects, which might be potentially useful in the prevention of ulcerative colitis [5]. Furthermore, phytoconstituents isolated from O*. baccatus*, including flavonoids, saponins, alkaloids, coumarins, and glucosinolates, exhibited anticancer, nematicidal, antiparasitic, and antioxidant potentials.

The Kingdom of Saudi Arabia (KSA) is famed for its traditional ethnomedical practices, which primarily include plants and plant derivatives, and for the abundance of medicinal plants that this country has been bestowed with [6]. In Saudi Arabia’s Hail region, people are increasingly using herbal medicine as an alternative treatment for a variety of chronic and acute diseases, including inflammations and infections. The consumption of herbal medicine has enormously increased due to the widespread belief that it has fewer side effects and provides faster healing, and consumption may become even more extensive once the phytochemical compounds of these traditional treatments have been identified and standardized [7]. *O. baccatus* is considered one of the most frequently used plants for treatment purposes in Hail and has been previously reported to possess a huge number of bioactive secondary metabolites. However, its medical benefits have not been fully validated, while investigations with regard to the plant’s potential biological activities against several diseases are limited and not well explored. This observation, together with native people’s consistent use of the plant, served as the impetus for this research, the purpose of which is to determine whether there are additional constituents that have the potential to be of pharmacological value.

Anticancer drugs and antibiotics are extremely important weapons in the fight against cancers and bacterial infections, respectively. Since their debut, these drugs have also significantly improved the health-related quality of life. Despite these benefits, however, many anticancer drugs and antibiotics result in toxic interactions in the body. Moreover, most anticancer drugs do not cure the disease that they target as they mostly just slow down disease progression, while on the other hand, the overuse of antibiotics leads to the development of drug-resistant microbial strains, which can make treatment more difficult. Therefore, it is absolutely necessary to conduct further research in order to discover more potent and less toxic anticancer drugs and antibiotics. In light of these considerations and in the context of our investigation on the biological activities of medicinal plants that are native to Saudi Arabia, the purpose of our study was to assess the anticancer and antibacterial activity of *O. baccatus* methanolic extracts in an attempt to develop it as an effective anticancer/antibacterial agent. Furthermore, we examined the chemical composition of this plant’s methanolic extracts *using LC-MS*. In general, the findings of this study highlight the importance of *O. baccatus* as a potential and useful source of natural anticancer and antibacterial agents, which can then be employed in the development of novel therapeutic chemicals in the future.

## 2. Materials and Methods

### 2.1. Collection and Preparation of Plant Materials

*O. baccatus* plant was collected during the spring season (February–March) from the region of Hail, Saudi Arabia. The plant material was identified by Dr. Naila H.Alkafei, (Department of Pharmacognosy and Phytochemistry, College of Pharmacy, University of Hafer Al Batin, Saudi Arabia). A voucher specimen (UOHCOP-G1) has been deposited at the College of Pharmacy, University of Hail, Hail, Saudi Arabia. The samples were dried naturally in the shade at room temperature (23–29 °C) for one to two weeks. The dried materials were ground to a fine powder to be used for extraction. The roots, stems, and fruits were dried separately, finely ground using an electric blender, and stored in plastic containers at room temperature and in darkness until required for use. The whole plant material was dried in the shade for one week and ground into a fine powder using an electrical blender.

### 2.2. Extraction

#### 2.2.1. *Ochradenus baccatus* (Flowers, Branches, and Roots)

The dried materials of the flower, branches, and roots were ground separately to a fine powder to be used for extraction. A total of 25 g of powder was extracted with 250 mL of MeOH at ambient temperature with the aid of sonication (4 × 15 min over at least a 24 h period) in a water bath. This mixture was filtered, and the resulting filtrate and residue were kept aside for further analysis.

#### 2.2.2. *Ochradenus baccatus* (Whole Plant)

Extraction was carried out by macerating the crude powder for 3 days in methanol, followed by filtration and evaporation at 35–40 °C. The dried extract was kept in a cold place until further use.

### 2.3. Liquid Chromatography–Mass Spectrometry (LC-MS) Analysis

Liquid chromatography–mass spectrometry (*LC-MS*) was used for the identification of our selected plant’s methanolic extracts (roots and branches). Liquid chromatography was performed using a Shimadzu ExionLC instrument (Kyoto, Japan), with mobile phase A containing 0.1% formic acid in water and mobile phase B containing acetonitrile. A GL-Science column with the dimensions (100 * 2.1) mm and 3 µm was used, while the solvents’ flow rate was 0.35 mL/min. The column oven temperature was 50 °C. The time program used for the analysis was 5% B (0–5 min), 5 to 95% B (5–30 min), and 95 to 5% B (30–40 min). Mass spectra were obtained using an X500R QTOF System (SCIEX, Redwood City, CA, USA) equipped with an electrospray ionization (ESI) system. The mass spectra were obtained in the positive and negative modes, and the scan type used was a full scan (SWATH Screening). Compound identification was performed by comparing the resulting mass spectra of the chemical compounds identified in the methanolic extracts with the National Institute of Standards and Technology (NIST) library database.

### 2.4. Antibacterial Activity

The plant extracts’ antibacterial potential was assessed using the broth microdilution susceptibility assay [8,9]. This assay was implemented in order to determine the plant extracts’ minimum inhibitory concentration (MIC) value, which was used to assess the extracts’ antibacterial activity. The methanolic extracts’ stock solutions in dimethylsulfoxide were prepared. Serial dilutions of the plant extracts’ stock solutions were prepared using sterile distilled water to obtain extract concentrations ranging from 1 mg/mL to 1.95 µg/mL. The extracts’ solutions were then transferred to 96-well microtiter plates. Using the McFarland No: 0.5 standard solution, the overnight-grown bacterial suspensions in double-strength Mueller-Hinton broth were standardized to 108 CFU/mL. Then, 100 L of each suspension of microorganisms was poured into each well. As a negative control, a well containing the broth without bacteria was employed. After incubation at 37 °C, the wells were inspected visually, whereby turbidity was indicative of bacterial growth. The minimum concentration of an *O. baccatus* methanolic extract that resulted in a clear well was defined as the MIC value for the extract against a particular bacterial strain. The bacterial strains used included *Escherichia coli* (ATCC 35218), *Escherichia coli* (ATCC 25922), *Salmonella typhimurium* (St) (ATCC 13311), and *Staphylococcus aureus* (Sa) (ATCC 25923). Chloramphenicol was used as the standard antibacterial agent (positive control).

### 2.5. Cell Culture

Colorectal cancer (HCT-116), breast adenocarcinoma (MCF-7), hepatocellular carcinoma (HepG2), and lung cancer (A-549) cells were maintained in RPMI media supplemented with 100 mg/mL of streptomycin, 100 units/mL of penicillin, and 10% of heat-inactivated fetal bovine serum under humidified, 5% (*v*/*v*) CO_2_ atmosphere at 37 °C.

### 2.6. Cytotoxicity Assay

Cell viability was assessed using the SRB assay. Aliquots of 100 μL of cell suspension (5 × 10^3^ cells) were placed into 96-well plates and incubated in complete media for 24 h. The cells were treated with another aliquot of 100 μL of the media containing drugs at various concentrations. After 72 h of drug exposure, the cells were fixed by replacing the media with 150 μL of 10% TCA and incubated at 4 °C for 1 h. The TCA solution was removed, and the cells were washed 5 times with distilled water. Aliquots of 70 μL of SRB solution (0.4% *w*/*v*) were added and incubated in a dark place at room temperature for 10 min. The plates were washed 3 times with 1% acetic acid and allowed to air dry overnight. Then, 150 μL of TRIS (10 mM) was added to dissolve the protein-bound SRB stain, and the absorbance was measured at 540 nm using a BMG LABTECH^®^-FLUOstar Omega microplate reader (BMG LABTECH-Ortenberg, Germany) [10].

### 2.7. Statistical Analysis

Statistical analysis was performed using the software Graphpad Prism, whereby related experiments were repeated three times and the resulting data were then analyzed via the software Graphpad Prism, version 8, San Diego, California. Analysis of the data via the software provided the mean and standard deviation of the repeated experiments. Significance differences between the results of the treated groups and that of the control group were also assessed via Graphpad Prism using two-way ANOVA, followed by Dunnett’s multiple comparison test. A result was considered significant if it possessed a *p*-value of less than 0.05.

## 3. Results

### 3.1. Antibacterial Evaluation of O. baccatus Extracts

The methanolic extracts of *O. baccatus* were evaluated for their antibacterial activity against *Escherichia coli* (E.coli 1), *Escherichia coli* (E.coli 2), *Salmonella typhimurium* (St), and *Staphylococcus aureus* (Sa) by using the broth microdilution antimicrobial susceptibility test to determine the extracts’ minimum inhibitory concentration (MIC) values. The concentrations used for the methanolic extracts in the assay ranged from 1 mg/mL to 1.95 µg/mL. The methanolic extracts of *O. baccatus* showed variable levels of activity against particular bacterial strains in the antibacterial MIC assay (Table 1). The highest antibacterial activity was exerted by the extract from the branches against *Escherichia coli* (ATCC 25922) and *Salmonella typhimurium (St)*, with MIC values of 15.6 µg/mL and 20 µg/mL, respectively. The *O. baccatus* whole plant’s methanolic extract and the flowers’ methanolic extract showed no significant activity against any of the investigated bacterial strains as they possessed MIC values of >1 mg/mL.

### 3.2. In Vitro Anticancer Evaluation of O. baccatus Extracts

**Figure 1:**
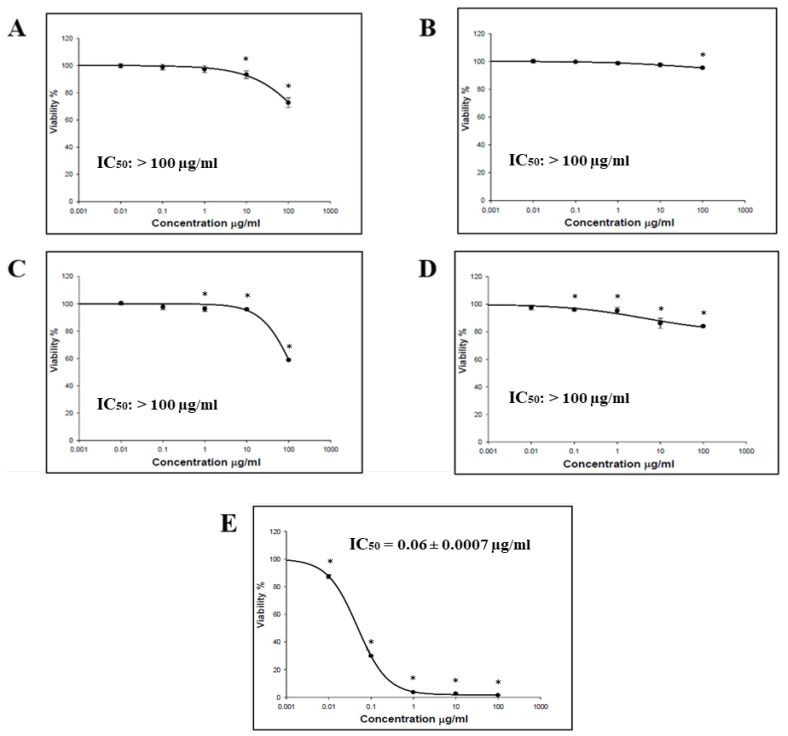
Representative dose–response plots demonstrating the effect of different *O. baccatus* methanolic extracts on HCT-116 cancer cell growth: (**A**) whole plant’s extract (**B**) flowers’ extract; (**C**) branches’ extract; (**D**) roots’ extract; and (**E**) doxorubicin. The experiment was repeated three times. Dots and error bars represent mean ± S.D. * *p* < 0.05 vs. control.

**Figure 2:**
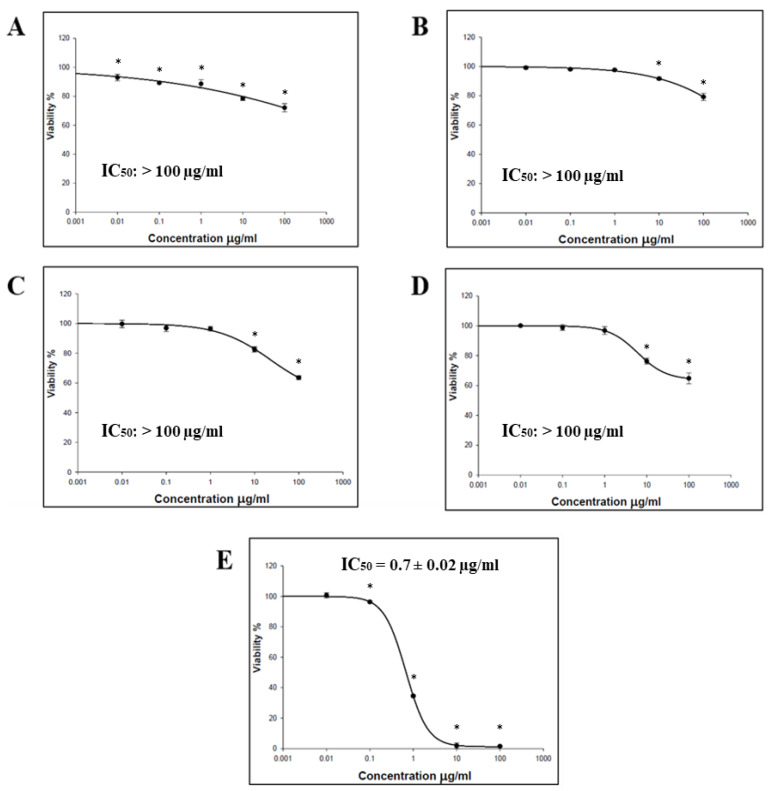
Representative dose–response plots demonstrating the effect of different *O. baccatus* methanolic extracts on HepG2 cancer cell growth: (**A**) whole plant’s extract; (**B**) flowers’ extract; (**C**) branches’ extract; (**D**) roots’ extract; and **(E**) doxorubicin. The experiment was repeated three times. Dots and error bars represent mean ± S.D. * *p* < 0.05 vs. control.

**Figure 3:**
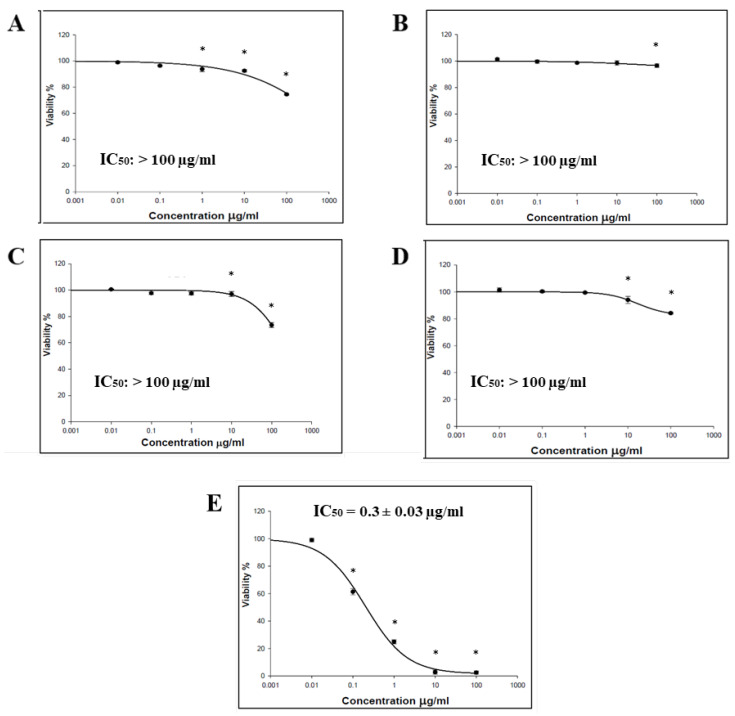
Representative dose–response plots demonstrating the effect of different *O. baccatus* methanolic extracts on MCF-7 cancer cell growth: (**A**) whole plant’s extract; (**B**) flowers’ extract; (**C**) branches’ extract; (**D**) roots’ extract; and (**E**) doxorubicin. The experiment was repeated three times. Dots and error bars represent mean ± S.D. * *p* < 0.05 vs. control.

**Figure 4:**
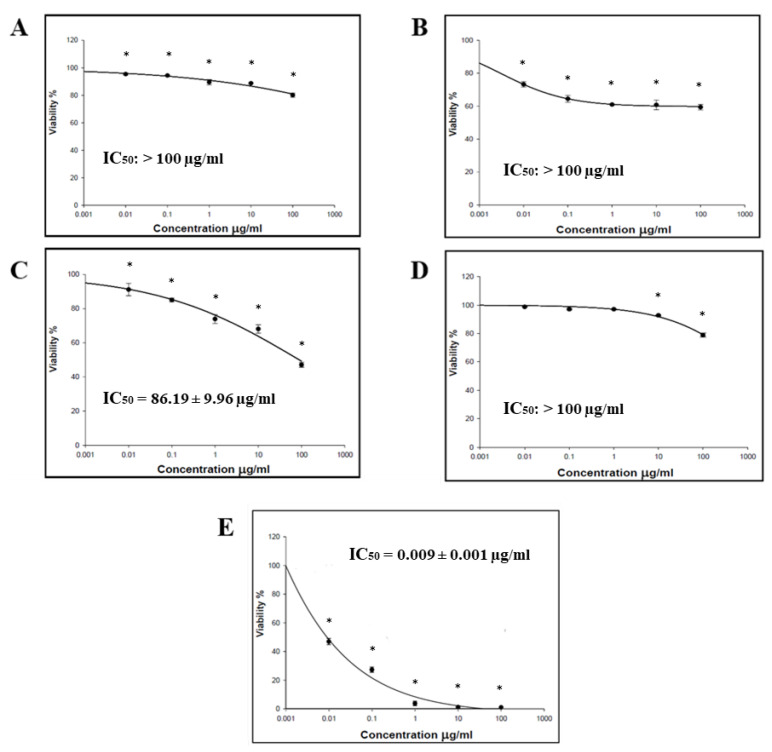
Representative dose–response plots demonstrating the effect of different *O. baccatus* ethanolic extracts on A549 cancer cell growth: (**A**) whole plant’s extract; (**B**) flowers’ extract; (**C**) branches’ extract; (**D**) roots’ extract; and (**E**) doxorubicin. The experiment was repeated three times. Dots and error bars represent mean ± S.D. * *p* < 0.05 vs. control.

The cytotoxic effect of the *O. baccatus* methanolic extracts (flowers, roots, branches, and whole plant) was performed using the SRB assay. Four cancer cell lines (HCT-116, MCF-7, HepG2, and A-549) were incubated with the extracts, and the status of cell growth was observed. No cytotoxic activity was generally demonstrated by all *O. baccatus* methanolic extracts against the examined cell lines, except for the *O. baccatus* branches’ methanolic extract, which exerted cytotoxic activity against A549 lung cancer cells with an IC_50_ value of 86.19 µg/mL (Figure 1, Figure 2, Figure 3 and Figure 4).

### 3.3. LC-MS Analysis of Biologically Active O. baccatus Methanolic Extracts

It is of extreme importance to investigate the phytochemical composition of bioactive plant extracts in order to better characterize them and to ensure that the obtained results are reproducible. The biologically active *O. baccatus* methanolic extracts, namely the extracts from the roots and branches, were subjected to *LC-MS* analysis in order to identify the chemical compounds that might be present in these extracts. A total of 8 and 13 major compounds were identified for both the roots’ and branches’ extracts, respectively, upon using the positive and negative ionization modes (Table 2 and Table 3).

## 4. Discussion

The present work was undertaken to find out the chemical composition and antibacterial/anticancer activities of *O. baccatus* methanolic extracts collected from the Hail region in Saudi Arabia. Therefore, different extracts were prepared from different *O. baccatus* plant parts, using methanol as a solvent, and biologically screened to demonstrate their potential antibacterial and anticancer activities. The methanolic extracts of *O. baccatus* were evaluated for their antibacterial activity against *Escherichia coli* (E.coli 1), *Escherichia coli* (E.coli 2), *Salmonella typhimurium* (S_t_), and *Staphylococcus aureus* (S_a_) by using the broth microdilution MIC antimicrobial assay at concentrations of 1 mg/mL–1.95 µg/mL (Table 1). The results showed that *O. baccatus* methanolic extract from the roots exerted antibacterial activity against *Escherichia coli-1*, *Escherichia coli-2*, *Salmonella typhimurium* (St), and *Staphylococcus aureus* (Sa) at MIC values of 250 µg/mL, 125 µg/mL, 62.5 µg/mL, and 500 µg/mL, respectively. Furthermore, the methanolic extract from *O. baccatus* branches inhibited the same bacterial strains’ growth at MIC values of 250 µg/mL, 15.6 µg/mL, 20 µg/mL, and 500 µg/mL, respectively. On the other hand, there was no antibacterial activity for the methanolic extracts *from O. baccatus* flowers and whole plant. Similar results have also been reported in previous studies [11,12,13,14]. Therefore, it could be clearly observed that the root and branch methanolic extracts demonstrated antibacterial activity, and the highest activity was exerted by the branch extract against *Escherichia coli* and *Salmonella typhimurium* (S_t_).

The cytotoxic activity of *O. baccatus* methanolic extracts (flowers, roots, branches, and whole plant) was performed using SRB assay. Four cancer cell lines (HCT-116, MCF-7, HepG2, and A-549) were incubated with the extracts, and the status of cell growth was observed after 72 h. No cytotoxic activity was generally demonstrated by the *O. baccatus* methanolic extracts against the tested cell lines, with the exception of A549 lung cancer cells whereby the extract from the branches exerted moderate activity against it, with an IC_50_ value of 86.19 µg/mL. It is important to note that this plant had been evaluated for its anticancer potential in a previous study and was discovered to be active in a human liver cancer cell line (HepG2) [15]. As a result, this is the first study to show that it may be effective against lung cancer as well. Such an IC_50_ value is considered significant for a plant extract, and the fact that it is selectively active against A549 cells is indicative of selectivity, which is a favorable property for any potential anticancer agent. This result is highly significant since further purification and isolation studies might yield highly potent compounds, and our study might provide a starting point for that.

The evaluation of the antibacterial and anticancer activities of the *O. baccatus* methanolic extracts showed that the extracts from the roots and branches were bioactive, while the extract from the flowers did not exhibit any biological activity. Therefore, an *LC-MS* analysis of the roots’ and branches’ extracts was conducted in order to identify their chemical constituents and to provide an explanation for their observed biological activities. This is the first study that investigated the chemical constituents of *O. baccatus* methanolic extracts. It is crucial to note that the *LC-MS* analysis of the *O. baccatus* methanolic extracts detected the presence of a wide variety of compounds, but most of them were present in low amounts and, thus, only the major compounds were considered. Moreover, it was not possible to know the identity of some *LC-MS* peaks by comparing their MS spectra with that of the NIST library database, but such peaks were few and the majority of the peaks were readily identifiable. Eight major compounds were identified in the *LC-MS* analysis of the roots’ extract (Table 2), and some of these compounds might have been responsible for the observed antibacterial activity. Azelaic acid (RT 8.62 min), ricinoleic acid (RT 13.39 min) and its derivatives, and liquiritigenin (RT 15.77 min) have been found to exert potent antibacterial activity [16,17,18]. Therefore, it is possible that these compounds might have been responsible for the observed antibacterial activity. Recent studies have also demonstrated the positive biological benefits of xylooligosaccharides such as 1,4-D-Xylobiose (RT 15.95 min), including their immunomodulatory and anticancer properties. Studies have shown that 1,4-D-Xylobiose’s anticancer effects are mediated via the modulation of glutathione homeostasis and TLR4 signaling, which impact cellular redox status. Therefore, it might be assumed that this chemical compound is also responsible for our plant’s carcinogenic properties [19]. The *LC-MS* analysis of the methanolic extract from *O. baccatus* branches identified 13 major compounds, and some of them are thought to be responsible for the extract’s antibacterial and anticancer activities (Table 3). Maslinic acid (RT 7.23 min), oleanolic acid (RT 8.44 min) and its derivatives, and lupenone (RT 16.06 min) have been found to possess antibacterial and anticancer activities [20,21,22,23,24,25]. β-amyrin (RT 7.23 min) and phytanic acid (RT 15.14 min) have also been found to demonstrate anticancer activity [26,27]. Moreover, several other studies established the genotoxicity and cytotoxicity of α-asarone (RT 7.57 min), and they discovered that it was toxic to the HepG2 cancer cell line [28]. Zymosterol (RT 15.95 min) has been demonstrated to possess antibacterial activity [29]. Therefore, the majority of the compounds that were identified in the bioactive extracts from *O. baccatus* roots and branches have been found to possess antibacterial and anticancer activities. These compounds are thought to play a role in the observed biological activities of our methanolic extracts via synergistic effects. Additionally, the vast majority of the chemicals that were extracted are also found in a wide variety of plant species. Two examples of this are the presence of azelaic acids in *Nicotiana tabacum* and the presence of ricinoleic acid in the seeds of *Ricinus communis*, which together make up nearly 90 percent of the seed oil’s total fatty acids. When it comes to the isolated active components that are responsible for cytotoxicity, these substances can be discovered in a wide variety of plants as well. One example of this is maslinic acid, which has been extracted from *Olea europaea* and *Salvia canariensis* and has been shown to have anti-inflammatory, antioxidant, and antineoplastic properties. β-amyrin may be found all over the natural world, and it has been extracted from a broad variety of plant sources, such as the wax that covers epicuticular structures. Moreover, α-asarone is a natural substance that can be discovered in many species, such as *Sphallerocarpus gracilis*, *Asarum hypogynum*, and others, for which data are accessible [30,31,32,33].

## 5. Conclusions

The phytoconstituents and crude extracts from various plant parts have been shown to exert a wide variety of biological actions in medical, laboratory, and research contexts. Although crude extracts from various plant parts have been used medicinally since prehistoric times, contemporary treatments can only be developed after rigorous research of their biological activity. The phytochemical content of *O. baccatus* is exceptionally high, and the plant contains a wide variety of phytochemicals with various biological activities. In this study, the antibacterial assay revealed that the methanolic extracts from *O. baccatus* roots and branches demonstrated varying degrees of activity against specific bacterial strains and were most active against *Salmonella typhimurium* (St) and *Escherichia coli*. However, the methanolic extracts from the whole plant and flowers did not demonstrate any antibacterial activity. Moreover, the results also showed that *O. baccatus* branches’ methanolic extract inhibited the growth of A549 lung cancer cells; however, the other methanolic extracts of the plant did not demonstrate any in vitro anticancer activity. This suggests that the branches’ methanolic extract demonstrates selectivity toward lung cancer cells (A549), and it would be interesting in the future to investigate the mechanism of action that is responsible for the observed cytotoxic activity against A549 cells. The *LC-MS* analysis identified several chemical constituents for the methanolic extracts from the roots and branches, and some of these chemical constituents are known for their bioactivity. Therefore, this study has proven the biological activities of *O. baccatus* extracts, and further studies could be conducted to isolate bioactive constituents and elucidate their mode of action, which might lead to the discovery of novel antibacterial and anticancer agents. It is also possible to use this study’s data to guide future in vivo studies on *O. baccatus* methanolic extracts, as the extracts themselves might be potent without the need for further isolation. The present work could serve as a starting point for researching the biological activities of various types of *O. baccatus* organic extracts. Documenting and analyzing traditional treatments is the most significant process of research on medicinal herbs. It is anticipated that this research will improve knowledge on the phytochemical and ethnopharmacological background of this genus and encourage additional research on *O. baccatus* and its primary phytoconstituents.

## Figures and Tables

**Table 1:** Antibacterial activity of the *O. baccatus* extracts.

Methanolic Extract	*E.coli 1*	*E.coli 2*	*St*	*Sa*
*Ochradenus Baccatus* (Whole plant)	>1 mg/mL	>1 mg/mL	>1 mg/mL	>1 mg/mL
*Ochradenus Baccatus* (Roots)	250 µg/mL	125 µg/mL	62.5 µg/mL	500 µg/mL
*Ochradenus Baccatus* (Branches)	250 µg/mL	15.6 µg/mL	20 µg/mL	500 µg/mL
*Ochradenus Baccatus* (Flowers)	>1 mg/mL	>1 mg/mL	>1 mg/mL	>1 mg/mL
Chloramphenicol	≤1.95 µg/mL	≤1.95 µg/mL	≤1.95 µg/mL	15.62 µg/mL

*E.coli 1*: *Escherichia coli* (ATCC 35218), *E.coli 2: Escherichia coli* (ATCC 25922), St: *Salmonella typhimurium.* (ATCC 13311), and Sa: *Staphylococcus aureus* (ATCC 25923). MIC (µg/mL) values of compounds.

**Table 2:** *LC-MS* analysis of methanolic extract of *O. baccatus* roots.

Compound	Retention Time (min)	Ionization Mode	Calculated Mass	Experimental Mass
**D-(+)-Trehalose (NIST)**	0.99	Negative	342.1162	341.1072 [M*−*H]^−^
**Azelaic acid (NIST)**	8.62	Negative	188.1049	187.0977 [M*−*H]^−^
**Ricinoleic acid** **(NIST)**	13.39	Negative	298.2508	297.2439 [M*−*H]^−^
***cis*** **-7-Hexadecenoic acid (NIST)**	15.07	Negative	254.2246	253.2173 [M*−*H]^−^
**Phytanic acid (NIST)**	15.17	Positive	312.3028	313.2733 [M+H]^+^
**Liquiritigenin (NIST)**	15.77	Negative	256.0736	255.2322 [M*−*H]^−^
**1,4-D-Xylobiose (NIST)**	15.95	Negative	282.0951	281.2476 [M*−*H]^−^
**Dodecyl sulfate (NIST)**	16.68	Negative	265.1479	265.1479 [M]^−^

**Table 3:** *LC-MS* analysis of methanolic extract of *O. baccatus* branches.

	Compound	Retention Time (min)	Ionization Mode	Calculated Mass	Experimental Mass
1	D-(+)-Trehalose (NIST)	0.92	Negative	342.1162	341.1071 [M*−*H]^−^
2	Maslinic acid (NIST)	7.23	Negative	472.3553	471.1889 [M*−*H]^−^
3	Alpha-Asarone (NIST)	7.57	Positive	208.1099	209.1549 [M+H]^+^
4	Alpha-Ionone (NIST)	7.79	Positive	192.1514	193.1599 [M+H]^+^
5	DELTA.2-*cis*-Eicosenoic acid (NIST)	7.87	Negative	310.2872	309.1368 [M*−*H]^−^
6	Beta-Amyrin (NIST)	7.98	Positive	426.3862	449.1068 [M+Na]^+^
7	2,4-Dimethylphenol (NIST)	8.05	Negative	122.0732	121.0296 [M*−*H]^−^
8	Oleanolic acid (NIST)	8.44	Negative	456.3603	455.1958 [M*−*H]^−^
9	Diphenylamine (NIST)	12.33	Positive	169.0891	170.0972 [M+H]^+^
10	2-Hydroxypalmitic acid (NIST)	14.82	Negative	272.2351	271.2277 [M*−*H]^−^
11	Phytanic acid (NIST)	15.14	Positive	312.3028	313.2733 [M+H]^+^
12	Zymosterol (NIST)	15.95	Positive	384.3392	385.3305 [M+H]^+^
13	Lupenone (NIST)	16.06	Positive	424.3705	425.3253 [M+H]^+^

## Data Availability

The data presented in this study are available from the corresponding authors upon request.
